# Acceptability and continuation of use of the subdermal contraceptive implant among adolescents and young women in Argentina: a retrospective cohort study

**DOI:** 10.1080/26410397.2023.2189507

**Published:** 2023-04-12

**Authors:** Daniel Maceira, Silvia Oizerovich, Gabriela Perrotta, Rodolfo Gómez Ponce de León, Ariel Karolinski, Natalia Suarez, Natalia Espinola, Sonja Caffe, Venkatraman Chandra-Mouli

**Affiliations:** a Professor, Department of Economics; Universidad de Buenos Aires, and Independent Researcher, National Council of Scientific and Technical Research (CONICET), Buenos Aires, Argentina; b Senior Researcher, Center for the Study of State and Society (CEDES), Buenos Aires, Argentina; c Board Member and Chair for The Americas, Health Systems Global (HSG). *Correspondence*: danielmaceira@cedes.org; d Professor, Faculty of Medical Sciences, Universidad Favaloro, Buenos Aires, Argentina; e President of the Ibero-American Network of Sexual and Reproductive Health Professionals, Buenos Aires, Argentina; f Member of the Scientific Committee of the Argentinian Pediatric and Adolescent Gynecology Society (SAGIJ) and Argentinian Medical Association for Contraception (AMAdA), Buenos Aires, Argentina; g Professor, Faculty of Psychology, Universidad de Buenos Aires, Buenos Aires, Argentina; i Sexual and Reproductive Health Regional Advisor at Pan-American Health Organization / World Health Organization (PAHO/WHO), Latin-American Center of Perinatology and Women’s Health (PAHO/CLAP), Montevideo, Uruguay; j Adjunct Professor School of Public Health of the University of North Carolina, Chapel Hill, NC, USA; k Advisor, Family, Health Promotion and Life Course; Coordinator of Family, Gender and Life Course, Pan-American Health Organization/World Health Organization (PAHO/WHO), Brasilia, Brazil Representation; l Professor, Universidad Nacional de Hurlingham, Community Health Institute, Buenos Aires, Argentina; m Teaching Assistant Professor, Department of Economics, University of Buenos Aires, Buenos Aires, Argentina; n Regional Advisor on Adolescent Health, Healthy Life Course Family, Health Promotion and Life Course PAHO/WHO, Washington, DC, USA; o Scientist, Adolescent Sexual and Reproductive Health, UNDP/UNFPA/UNICEF/WHO/World Bank Special Program of Research, Development and Research Training in Human Reproduction (HRP), Department of Sexual and Reproductive Health and Research, World Health Organization, Geneva, Switzerland

**Keywords:** Adolescence, pregnancy, public policy, subdermal implant, satisfaction, adherence

## Abstract

A new public policy was instituted in Argentina for free distribution of subdermal contraceptive implants to women aged 15–24 years old in the public healthcare system. The objective of this study is to determine the extent to which this population adhered to the implant, as well as predictors of continuation. The retrospective cohort study was based on a telephone survey of a random sample of 1101 Ministry of Health-registered implant users concerning the continuation of use, satisfaction with the method and side-effects, and reasons for removal. Descriptive statistics and multivariate regression analysis were used to explore the association between adherence and having received contraceptive counselling, satisfaction, and side effects. We found high levels of adherence (87%) and satisfaction (94%). Common reported side effects were amenorrhoea or infrequent bleeding, perceived weight gain, increased menstrual bleeding and headaches. Multivariate regression analysis indicates that, among adolescents, having received contraceptive counselling increased comfort, while frequent bleeding at six months hindered trust. Participants who had a history of a prior delivery or who had themselves primarily chosen the method were less likely to request the removal of the implant. Our results support the public policy of free implant distribution in the public health sector. This is a sustainable public policy that contributes to equity and access to effective contraception. It is appropriate for adolescents and young women and will also reduce unintended pregnancies. Our results suggest that counselling patients is key prior to insertion of the implant, as it improves acceptability and continuation.

## Résumé

En 2014, en Argentine, une nouvelle politique publique a institué la distribution gratuite d’implants contraceptifs sous-cutanés aux femmes âgées de 15 à 24 ans dans le système de santé publique. L’objectif de cette étude était de déterminer dans quelle mesure cette population a adhéré à l’implant, ainsi que les indicateurs de la poursuite de cette méthode. L’étude de cohorte rétrospective était fondée sur une enquête par téléphone auprès d’un échantillon aléatoire de 1101 utilisatrices d’implant enregistrées auprès du Ministère de la santé concernant la poursuite de l’utilisation, la satisfaction quant à la méthode et les effets secondaires, ainsi que les raisons du retrait. Des statistiques descriptives et une analyse de régression multivariée ont été utilisées pour explorer l’association entre l’adhérence et l’obtention de conseils contraceptifs, la satisfaction et les effets secondaires. Nous avons observé des niveaux élevés d’adhérence (87%) et de satisfaction (84%). Les effets secondaires fréquemment signalés étaient une aménorrhée ou des saignements peu fréquents, une prise de poids perçue, des saignements menstruels accrus et des maux de tête. L’analyse de régression multivariée indique que, chez les adolescentes, le fait d’avoir reçu des conseils contraceptifs a augmenté leur aisance, alors que des saignements fréquents à six mois ont sapé la confiance. Les participantes qui avaient déjà accouché ou avaient elles-mêmes choisi la méthode en amont avaient moins de probabilités de demander le retrait de l’implant. Nos résultats étayent la politique de distribution gratuite d’implants dans le secteur de la santé publique. C’est une politique publique durable qui contribue à l’équité et à l’accès à une contraception efficace. Elle est adaptée aux adolescentes et aux jeunes femme et réduira également les grossesses non désirées. Nos conclusions montrent que le conseil aux patientes est essentiel avant l’insertion de l’implant car il améliore l’acceptabilité et la poursuite de cette méthode contraceptive.

## Resumen

En 2014, se instituyó una nueva política pública en Argentina para la distribución gratuita de implantes anticonceptivos subdérmicos a mujeres entre 15 y 24 años en el sistema de salud pública. El objetivo de este estudio es determinar en qué medida esta población se adhirió al implante, así como los predictores de continuación. El estudio retrospectivo de cohorte se basó en una encuesta telefónica de una muestra aleatoria de 1101 usuarias de implantes inscritas en el Ministerio de Salud sobre la continuación de uso, la satisfacción con el método y los efectos secundarios, y las razones para la extracción. Se utilizaron estadísticas descriptivas y análisis de regresión multivariable para examinar la asociación entre la adherencia al método y haber recibido consejería anticonceptiva, la satisfacción y los efectos secundarios. Encontramos altos niveles de adherencia (87%) y satisfacción (94%). Los efectos secundarios comunes informados fueron amenorrea o sangrado infrecuente, percepción de aumento de peso, aumento del sangrado menstrual y dolores de cabeza. El análisis de regresión multivariable indica que, entre adolescentes, haber recibido consejería anticonceptiva aumentó la confianza con el método, mientras que el sangrado frecuente a los 6 meses obstaculizó la confianza. Las participantes que tenían antecedentes de un parto anterior o que habían elegido el método por sí mismas principalmente eran menos propensas a solicitar la extracción del implante. Nuestros resultados apoyan la política pública de distribución gratuita de implantes en el sector de salud pública. Es una política sostenible que contribuye a la equidad y al acceso a anticoncepción eficaz. Es adecuada para adolescentes y mujeres jóvenes y reducirá las tasas de embarazos no intencionales. Nuestros resultados indican que brindar consejería a las pacientes es clave antes de la inserción del implante, ya que mejora su aceptación y continuación.

According to World Health Organization (WHO) estimates, 16 million adolescents aged 15–19 and one million younger than 15 give birth each year.^1^ An additional three million adolescents have unsafe abortions each year.^1^ In Latin America and the Caribbean (LAC), the fertility rate for 15–19-year-olds is 63/1,000, much higher than the 42.5/1,000 reported globally; this rate is higher only in Africa (95/1,000). In Asia, North America, and Oceania, the rates vary between 18.9 and 27.9/1,000, while in Europe they are below 12.7/1.^2^ These regional estimates reflect inequities in access to healthcare services and to contraceptives,^3^ as one of a complex set of factors, which also results in large inequities in terms of unintended pregnancies.^4^ Argentina’s adolescent fertility rate has declined from 65.7/1000 live births in 15–19 years old in 2011 to 40.7 in 2019. While there has been progress, more needs to be done.

In Argentina, 91,586 live births to adolescents aged 15–19 were recorded in 2017 (13% of the total number of births). There were also 2,493 live births to adolescents younger than 15 (0.3% of all births nationally).^5^ According to statistics from the Ministry of Health and records of the Perinatal Information System (SIP), which gathers information from around 70% of the public maternity wards in the country, 7 out of 10 adolescents who gave birth in 2019 reported that their pregnancies were unplanned.^6^ Among girls under the age of 15, 8 out of 10 pregnancies were unintentional, and to a large extent, these have been the consequence of sexual abuse and violence.^6^ Further, of the 202 maternal deaths recorded in 2017 by the Ministry of Health, 26 (12.8%) were among adolescents (two were due to abortion complications).^5^

Unintended adolescent pregnancy has multiple determinants, including lack of access to information and to effective contraception, incorrect use of contraception, gender, and societal barriers.^7^ In the last few years, interest in long-acting reversible contraceptives (LARCs)* has increased. These methods, which include the subdermal implant, are highly effective.^7,8^ Their primary advantage is that they do not depend on the user to perform repeated actions to guarantee correct use of the method,^8,9^ which is particularly relevant in vulnerable populations and those with limited access to health services.

Large-scale projects have been developed in the last few years to increase access to LARCs, such as the CHOICE Project^10^ and the Colorado Family Planning Initiative in the United States,^11^ which have included adolescents as their targeted groups. Evaluations of these projects found high rates of acceptability (75%) and continuation (77% at 2 years) after training healthcare teams to promote the provision of LARC methods. They also found that users of short-acting methods were more than 20 times more likely to have an unintended pregnancy than those who used a LARC. A recent systematic review exploring the continuation of LARCs among adolescents found that across twelve studies, including high-income and middle-income countries, continuation rates for the implant at one year were very high (84% pooled analysis).^12^

## The context of unintended adolescent pregnancy prevention in Argentina

The legal framework for sexual and reproductive health and rights in Argentina has led to the development and execution of several very important public policies and programmes nationwide, including those focused on reducing unintended pregnancies among adolescents. Among these is the creation of the national sexual health and family planning programme (PNSSyPR, from the name in Spanish) by Law 25,673 in 2002. This law guarantees access to information, counselling, public services at the primary level and referral when needed, as well as the free provision of contraceptive methods to guarantee access and coverage to the most vulnerable populations nationwide. The Division of Sexual and Reproductive Health offers technical assistance and trainings for healthcare providers and leaders, generates clinical guidelines and protocols, monitors actions in the different provinces and regions and carries out other actions together with other related government bodies to inform and educate the general population.

According to world population projections by the United Nations in 2019, the fertility in Argentina is below the world average but above the regional average.^2^ In the five-year period 2015-2020, the global fertility rate was 2.5 children per woman worldwide, 2.0 in LAC, and 1.9 in South America, while in Argentina women had 2.3 children per woman.^13,14^ This trend has been heterogeneous by regions and age groups, influenced by great geographical, social, political, and economic disparities.

Despite varied efforts and supplies distributed, Argentina had the LAC region’s highest adolescent fertility rate in 2011 (69.6/1000). In subsequent years, the rate began to decrease but did so more rapidly only after 2014.^15^

Coincidently in 2014, the Ministry of Health included contraceptive implants within the basic basket of contraceptive supplies of the PNSSyPR, specifically in the seven regions of Argentina with the highest adolescent fertility rates.^16,17^ The target population for this programme was 15–19-year-old adolescents who had already been pregnant (pregnancy ending in childbirth or abortion) in the year preceding the insertion of the implant. One of the main pillars of this policy is counselling, which means providing information about available methods, their characteristics and possible side effects. This is a mandatory standard of care, and is usually accompanied by supportive educational material, including fliers and posters.^16^

In 2015, the programme was expanded to cover the entire country, for women up to 24 years old using the public health system, regardless of their pregnancy history. The goal of this programme was to increase the use of a modern and safe contraceptive method and to decrease the barriers to accessing effective contraception among low-income women.^16^

While acceptability of contraceptives, including oral contraception,^18^ emergency contraception,^19^ and the intrauterine device (IUD) is high country-wide,^20–22^ to date a thorough national-level assessment of the acceptability and continuation rates of contraceptive implants has not been undertaken. The only national survey on sexual and reproductive health was completed in 2013 (Argentina, 2011–2012 survey) and the implants were not yet available in the public health system at that time.^22^

The objective of this study was to analyse the acceptability and continuation of contraceptive implants among adolescents and young women in Argentina, over the period January-December 2017. We analysed acceptability by examining users’ satisfaction with the method, which we measured in terms of trust and comfort, and continuation by examining whether users requested the removal of the method, as well as factors that contributed to the continuation or discontinuation of the method.

## Methods

### Study design, source of information and population

We used a retrospective cohort study design for this evaluation. We administered a phone survey to young women aged 15–24 years old who had received an implant through the Argentinian public health system in 2015. The Ministry of Health designed and implemented the survey, which was modified after piloting with 20 respondents and establishing that the questions were precise and could be well understood. The survey collected information about demographic and general reproductive health characteristics of the users, as well as information specific to implant use, such as bleeding patterns, side effects, and satisfaction, including reason for implant removal if applicable.

This study was carried out through the Ministry of Health, as part of the regular care follow-up and evaluation. We identified the women for our evaluation through the national database of all reported implant insertions done through the public health system, considering women for whom informed consent and telephone contact information were available. The follow-up was carried out in 2017, two years after the placement of the implants in 2015. A random probability sample was used, stratified by geographical region and age group. The phone survey was administered as a follow-up, which is a part of the regular standard operating procedure for follow-up care. From a total of 15,440 women aged 15–24 years old who received the implant in 2015, we randomly selected 1,951 using a sample size calculation of 10%. Each woman was contacted by trained SRH interviewers over the phone, and in the case they could not be reached (due to a change in phone number or unanswered calls) in three calls, they were replaced with another of the same age group, until we reached the desired sample size for each geographical region, obtaining a final sample size of 1001 women, proportionally distributed by age and region.

This study used the same contraceptive information, counselling and service provision procedures that are used routinely by the Ministry of Health. This includes the follow-up call. The database was anonymised, and women’s identity was protected. The Ethical Committee of the Durand Hospital in Buenos Aires (version number 1.01, December 2016) and the PAHO Ethical Committee (PAHOERC, approval number 12-0060, 14 February 2017) approved this study.

### Dependent variables

The study-dependent variables were dichotomous variables that measured if the woman felt comfortable with the implant and trusted in the implant (yes / no), according to age group (15-19 or 20-24). Comfort variable was constructed based on the question referring to the degree of contentment (from 1 to 10, where 10 represents the highest degree of satisfaction) in using the method, considering the administration technique and duration. The comfort with the implant variable takes the value 1 if the reported degree of comfort was greater than and equal to 7, and 0 if the reported degree of comfort was less than 7. The trust in the implant variable was constructed in a similar way, based on the question about the degree of confidence (from 1 to 10, where 10 represents the highest degree of satisfaction) in the capacity of the method to prevent pregnancy. The variable takes the value 1 if the person surveyed reported a grade greater than or equal to 7, and 0 otherwise. Following international recommendations, continuation was defined as choosing to maintain the implant for three years. At the time of our evaluation, we assessed the discontinuation of the method at six months and one year after insertion.

### Independent variables

The study included the following variables. Education level was divided into three groups: < 8 years of study, 8-12 years, and more than 12 years. In the econometricmodels, the variable was converted into a dichotomous variable, taking the value 1 if the person had up to and equal to 12 years of education, and 0 if they had more. Other variables were: *employed variable (employed / unemployed), history of prior deliveries (yes/no), history of prior abortions (yes/no), user´s decision to have implant inserted (yes/no), recalled receiving counselling (yes/no).* The variables of side effects reportedwere constructed as dichotomous variables (yes/no) based on the questions referring to how menstruation was six months after the implant was placed (amenorrhoea at six months, infrequent bleeding at six months, frequent bleeding at six months, prolonged or abundant bleeding at six months), and the changes perceived at the beginning of the implant placement. If the person surveyed reported one or more of these events, the variable takes the value 1 (yes), and 0 (no) otherwise.

### Statistical analysis

We used descriptive statistics to present absolute and relative frequencies for continuation, satisfaction, side effects, and reasons for requesting implant removal. We analysed data for the entire study population and disaggregated by age group: 15–19 years (adolescents) and 20–24 years (youth). In terms of geography, we grouped provinces into regions for the purposes of data analysis.

In all cases, the margin of error between the regional and sample distribution of women with implants does not exceed 3%, accounting for representativeness.

Multivariate regression analysis was conducted using a logit model to explore the association between women’s satisfaction, measured as comfort and trust, with using the implant. For the association with comfort and trust, we used two different models for each age group that included the following variables which proved significant in our univariate analysis: education, employment status, history of prior childbirths or abortions, whether she chose the method, whether she recalled receiving contraceptive counselling, prevalence of known side effects from the method and other perceived symptoms associated to method used. The construction of these variables was explained previously.

We used Student’s *t*-test to test for significant mean differences among adolescents and youth. Statistical significance is reported at *p* < 0.05 for all descriptive statistics and regressions. We conducted all data integration, processing and analysis using Stata® v14.2 (Stata Corporation, College Station, Texas, USA).

## Results

### Sample and implant-related characteristics

From the 1001 women included in our analysis, 576 were adolescents (15-19 years old) and 525 were young women (20–24 years old) (Table 1). Around one-third of participants resided in the Metropolitan Area of Buenos Aires, and almost 70% had 8–12 years of education. Less than 50% of participants were working, but this percentage was lower among the adolescents. In terms of obstetrical history, 73.1% of participants reported at least one prior abortion or childbirth (almost 65% of adolescents and more than 80% of young women).

**Table 1.** Description of the study population of women with subdermal contraceptive implants according to age group (*N* = 1101)

Population characteristics	Age: 15–19 years old	Age: 20–24 years old	Total number
	*N* = 576, *N* (%)	*N* = 525, *N* (%)	*N* = 1101, *N* (%)
**Place of residence**^a^			
Metropolitan area of Buenos Aires	186 (32.3)	174 (33.1)	360 (32.7)
Cuyo	130 (22.6)	78 (14.9)	208 (18.9)
Centre	78 (13.5)	25 (4.8)	103 (9.4)
Northeast	81 (14.1)	114 (21.7)	195 (17.7)
Northwest	75 (13.0)	124 (23.6)	199 (18.1)
Patagonia	26 (4.5)	10 (1.9)	36 (3.3)
**Education** (maximum level achieved, in years)			
<8 years	26 (4.5)	28 (5.3)	54 (4.9)
8–12 years	435 (75.5)	323 (61.5)	758 (68.9)
>12 years	114 (19.8)	171 (32.6)	285 (25.9)
Missing	1 (0.2)	3 (0.6)	4 (0.4)
**Employment status**			
Employed	207 (35.9)	241 (45.9)	448 (40.7)
Unemployed	369 (64.1)	282 (53.7)	651 (59.1)
Missing	0	2 (0.4)	2 (0.2)
**Obstetrical history**			
No prior pregnancies	146 (25.3)	35 (6.7)	181 (16.4)
History of abortion	57 (9.9)	58 (11.0)	115 (10.4)
History of prior vaginal or caesarean delivery	373 (64.8)	432 (82.3)	805 (73.1)
**Decision to have the implant inserted**	** **	** **	** **
User	479 (83.2)	433 (82.5)	912 (82.8)
Others (healthcare professional or other)	31 (5.4)	12 (2.3)	43 (3.9)
Missing	66 (11.5)	80 (15.2)	146 (13.3)
**Recall receiving counselling**	** **	** **	** **
Yes	390 (67.7)	372 (70.9)	762 (69.2)
No	120 (20.8)	73 (13.9)	193 (17.5)
No data	66 (11.5)	80 (15.2)	146 (13.3)
**Requested implant removal**	65 (11.3)	79 (15.1)	144 (13.1)
6 months	19 (3.30)	32 (6.1)	51 (4.6)
1 year	39 (6.8)	41 (7.8)	80 (7.2)

^a^ For place of residence, regions are defined as: Buenos Aires metropolitan region, Centro (Córdoba, La Pampa, Entre Ríos, Santa Fe, Buenos Aires except metropolitan region), Cuyo (Mendoza, San Juan y San Luis), Northeast (Misiones, Corrientes, Chaco, Entre Ríos y Formosa), Northwest (Jujuy, Salta, Tucumán, Catamarca, La Rioja y Santiago del Estero), Patagonia (Neuquén, Río Negro, Chubut, Santa Cruz y Tierra del Fuego, y Antártida e Islas del Atlántico Sur).

Approximately 70% of women had a clear recall of having received contraceptive counselling prior to the insertion of the implant. (This is part of the standard operating procedures set down by the Ministry of Health). In terms of the decision to have an implant installed, 82.8% of women declared to have decided by themselves to have the implant inserted. The remaining 17.2% declared that the primary decision was taken by either a doctor, other professionals, family members or partners.

**Figure 1.**
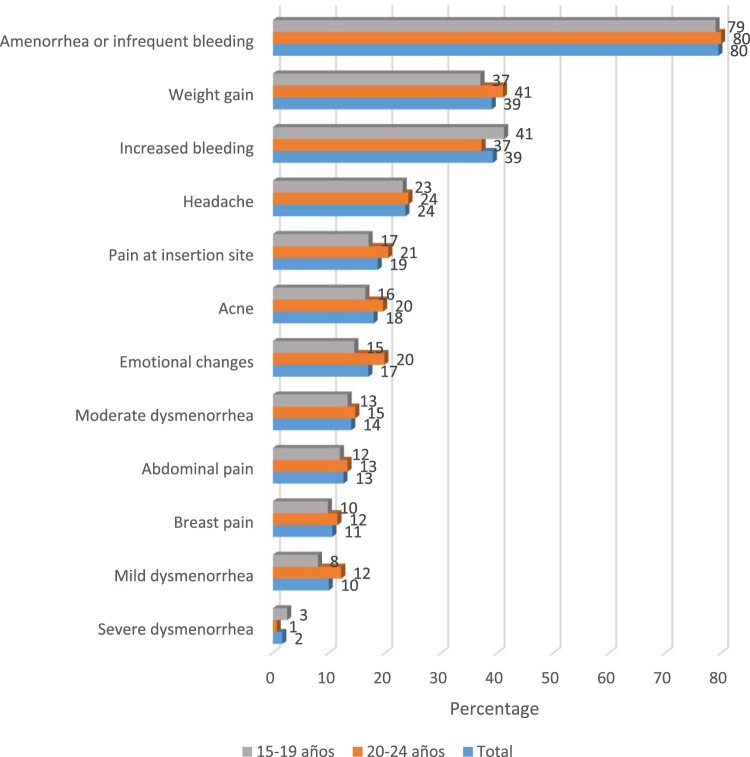
Proportion of women who reported side effects after implant insertion, according to the age group (*N* = 956)

The most common side effect, reported by approximately 80% of participants, was amenorrhoea or infrequent bleeding. Other side effects, such as weight gain, were mentioned by less than half of the sample (Figure 1).

### Method acceptability

Acceptability of the subdermal contraceptive implant among adolescents and young women was examined through users' satisfaction with the method, measured in terms of trust and comfort.

We found a high level of acceptability of the implant as measured through satisfaction (including trust and comfort with the method) of over 90%. The average was 9.4/10 for both age groups and for both measures of satisfaction.

After controlling for potential confounders, clearly recalling having received contraceptive counselling was positively associated with comfort with the method among adolescents (1.72 OR, 95% CI 1.10–2.68), while breast pain (0.48 OR, 95% CI 0.26–0.88) and headaches (0.63 OR, 95% CI 0.41–0.98) were negatively associated with comfort. On the other hand, the odds of reporting confidence in the method among young women were positively associated with working (1.63 aOR, 95% CI 1.05–2.52) and negatively associated with higher levels of education (0.50 aOR, 95% CI 0.33–0.75) and having had a prior abortion (0.49 aOR, 95% CI 0.25–0.94). Frequent bleeding at six months was negatively associated with trust among adolescents (0.46 aOR, 95% CI 0.23–0.90) while weight gain was positively associated with trust (1.55 aOR, 95% CI 1.01–2.38). (See Table 2 for the results of the multivariate regressions).

**Table 2.** Satisfaction with the implant according to comfort and trust, according to characteristics of women and side effects reported (*N* = 1101)

Variables included	Comfort with the implant				Trust in the implant			
	Adolescents 15–19 years old		Youth 20–24 years old		Adolescents 15–19 years old		Youth 20–24 years old	
	aOR	95% CI	aOR	95% CI	aOR 95% CI		aOR 95% CI	
***Demographic characteristics***	*** ***	*** ***	*** ***	*** ***	*** ***	*** ***	*** ***	*** ***
Mandatory education (≤12 years)	0.81	0.56–1.17	0.81	0.56–1.16	0.86	0.60–1.24	0.50*	0.33–0.75
Employed	0.99	0.68–1.44	0.86	0.56–1.31	1.41	0.94–2.11	1.63*	1.05–2.52
History of prior deliveries	1.48*	1.00–2.19	0.69	0.38–1.25	1.37	0.94–2.01	1.05	0.63–1.76
History of prior abortions	1.38	0.74–2.55	1.29	0.65–2.57	1.34	0.72–2.47	0.49	0.25–0.94
***User's decision to have implant inserted***	1.04	0.45–2.42	2.85	0.44–18.54	1.14	0.53–2.46	1.68	0.35–8.03
***Recalled receiving counselling***	1.72*	1.10–2.68	1.19	0.69–2.05	1.41	0.89–2.23	1.08	0.59–1.99
***Side effects reported***	* *	* *	* *	* *	* *	* *	* *	* *
Amenorrhoea at six months	1.01	0.63–1.62	0.91	0.51–1.62	1.21	0.75–1.95	0.93	0.51–1.69
Infrequent bleeding at six months	0.99	0.60–1.65	0.81	0.43–1.51	1.30	0.78–2.17	1.29	0.70–2.41
Frequent bleeding at six months	0.61	0.33–1.16	0.70	0.30–1.62	0.46*	0.23–0.90	0.78	0.33–1.88
Prolonged or abundant bleeding at six months	0.89	0.52–1.51	0.84	0.43–1.61	1.30	0.75–2.25	0.77	0.39–1.50
Breast pain	0.48*	0.26–0.88	0.57	0.30–1.06	0.90	0.50–1.62	0.96	0.51–1.81
Acne	1.09	0.64–1.86	0.70	0.44–1.12	0.81	0.47–1.39	1.42	0.84–2.38
Headache	0.63*	0.41–0.98	0.64	0.38–1.09	1.12	0.70–1.79	1.15	0.65–2.03
Emotional changes	1.06	0.59–1.92	0.69	0.42–1.13	0.59	0.32–1.11	0.58	0.33–1.00
Abdominal pain	0.74	0.42–1.31	1.07	0.56–2.03	1.41	0.69–2.89	0.73	0.34–1.56
Pain at time of insertion	0.90	0.55–1.47	0.68	0.42–1.09	0.73	0.45–1.18	1.05	0.62–1.77
Dysmenorrhea	1.27	0.60–2.72	1.70	0.88–3.29	1.12	0.58–2.16	1.35	0.65–2.79
Perceived weight gain	1.06	0.70–1.59	0.68	0.44–1.06	1.55*	1.01–2.38	0.98	0.63–1.53

Notes: aOR: adjusted odds ratio; CI: confidence interval; *p < 0.05. Adjusting for: age, years of education, employment status, obstetric history, whether the woman chose the implant herself, recalls having received counselling, and reported side effects (amenorrhoea, irregular bleeding, breast pain, acne, headaches, emotional changes, abdominal pain, pain at insertion, weight gain). Reference for all variables was YES.

### Method continuation

Continuation of the subdermal contraceptive implant among adolescents and young women was examined through users' method removal requests, as well as analysis of the factors associated with the continuation or discontinuation of the method.

**Figure 2.**
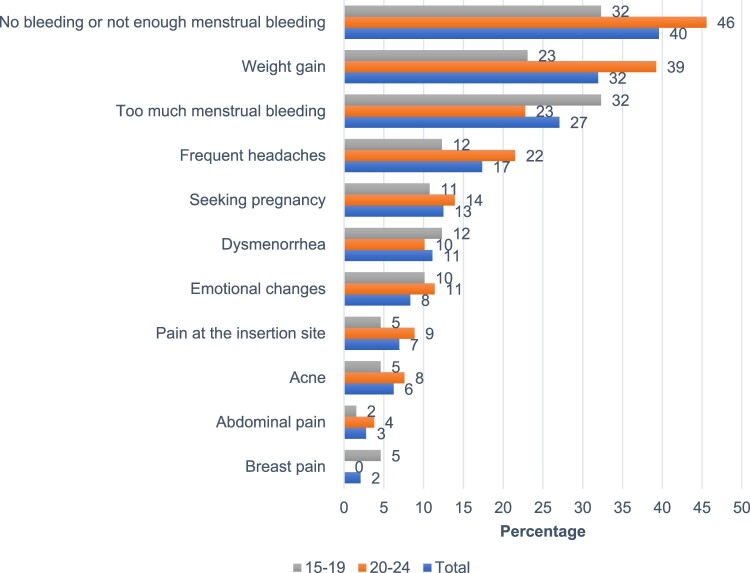
Reasons reported by women for having the implant removed, total and by age range (*N* = 144)

Most women analysed retained the method for the entire study period, with only 11% and 15% of adolescents and young women, respectively, requesting removal of the implant in the first year. A minority of participants (144/1001, 13%) requested implant removal. Of these 144 women, 22 requested the implant to be removed at six months (15%) and an additional 111 by the end of the first year (total 91%, 133/144). Only 10 women (7%, 10/144) requested removal after the one-year mark. 11.9% of young women who had contraceptive implants placed, requested their removal in the first year, and 1.2% of them did so in the second year. The two most common reasons for requesting implant removal were the absence or decrease of menstrual bleeding (40%) and weight gain (32%) (Figure 2).

After controlling for potential confounders, having had prior childbirths or having chosen to request the method herself was negatively associated with requesting the removal of the implant among adolescents (0.47 aOR, 95% CI 0.28–0.82; and 0.38 aOR, 95% CI 0.16–0.92). Among young women, working was positively associated with requesting the removal of the implant (1.74 aOR, 95% CI 0.85–3.56) (Table 3).

**Table 3.** Association between having requested implant removal and participant characteristics (*N* = 1101)

Variables included	Adolescents 15–19 years old		Youth 20–24 years old	
	aOR	95% CI	aOR	95% CI
***Demographic characteristics***	* *	* *	* *	* *
Mandatory education (up to 12 years)	0.96	0.55–1.67	1.28	0.81–2.00
Employed	0.90	0.51–1.58	*1.74	1.05–2.87
History of prior deliveries	*0.47	0.28–0.82	0.87	0.47–1.62
History of prior abortions	0.45	0.15–1.38	1.74	0.853.56
***Having themselves asked for the implant***	*0.38	0.16–0.92	0.38	0.13–1.13
***Clearly recalls having received contraceptive counselling***	2.19	0.99–4.84	0.76	0.40–.142

Notes: aOR: adjusted odds ratios; CI: confidence interval; *p < 0.05. Adjusting for: whether the woman had up to 12 years of education, employment status, obstetric history, whether the woman asked for the implant herself, and recalls having received counselling. Reference for all variables was YES.

The main cause of requesting the removal of the implant was the presence of two side effects, i.e. no bleeding or decreased bleeding, and weight gain. Young women in the 20–24 years age group and those who were working were more likely than younger ones and those who were not working to ask for the implants to be removed. Adolescents and young women who themselves asked for the implants (rather than have health workers or family members propose them), those who clearly recalled receiving counselling, and those who were well aware of potential side effects were more likely to retain the implants longer.

## Discussion

We aimed to describe and analyse the acceptability and continuation of the use of contraceptive implants provided to young women aged 15–24 years through the public health system in Argentina. We also sought to understand the factors that contributed to continuation and discontinuation of the method.

We found a high level of acceptability of the implant as measured through satisfaction (including trust and comfort with the method). Overall continuation rates at one year in our study (86%) were similar to that found in a systematic review of mainly US studies analysing continuation of LARCs at 12-months post-insertion (84%).^12^ Further, the proportion of women requesting removal of the implant within the first year of insertion was in the lower range of those in other studies (11.9% in our study vs 11.8–33% in others).^23–26^ Requests for removal in the second year were also very low (1.2%), in line with the findings of other studies.^23–29^ Moreover, additional studies have shown – as our study did – that high one-year continuation rates can be achieved through intensive counselling and consideration for patient preference.^28–31^

We also found that adolescents 15–19 years old were less likely to request implant removal (only 11% did) compared to their 20–24 years old counterparts (15%), which may be associated with lower agency of young women at requesting implant removal. The overall results suggest that the implant is a useful strategy to prevent unintended pregnancies among adolescents and young women.

Our finding regarding the acceptability of the implant and satisfaction with its use is consistent with the literature. We also found that women receiving counselling were more likely to report satisfaction with it, similar to a study in the United Kingdom which found that satisfaction and continuation were higher among women who had received information about side effects, including amenorrhoea and irregular bleeding.^29^ Another study conducted in the United States also found that women who were more familiar with the method and its effects were more likely to find it acceptable.^24^

We draw three conclusions from the findings of our study placed within the context of existing evidence. First, implants are an acceptable means of contraception for adolescent girls and young women in Argentina. Second, the information and counselling support provided to adolescent girls and young women by public health sector delivery staff contributed to the high level of satisfaction with the implant. And third, with targeted support for those girls and women who experience side effects, a continuation of the method could be further increased.

Our study provides clear programmatic guidance for the provision of contraceptive implants to adolescents and young women through the public health system to reduce unplanned pregnancies and improve women’s contraceptive autonomy. In Argentina, efforts should be made to sustain the nationwide implant distribution and delivery programme and to further strengthen the provision of counselling support to young women. Given that most deliveries in Argentina are facility-based (99.5%),^5^ implants could be an integral part of the variety of methods offered post-partum and post-abortion. Our call for active promotion is very much in line with the recommendations by Saavedra-Avendano et al. and Darney et al., based on studies in Mexico.^32,33^ Beyond Argentina, findings from our study can be used to inform public health policy aimed at adolescents’ health and well-being, to achieve the SDGs and the Global Strategy for Women, Children, and Adolescents 2016–2030.^34,35^

Two key research questions remain. On the one hand, we need to better understand the quality of the counselling provision, as well as to develop and test approaches to further improve it in different contexts in Argentina and elsewhere. On the other hand, we need to understand the socio-economic attributes of the young women who continued to use the implant and those who did not, to then consider what actions could be taken for each of these groups in a more targeted fashion.

Our study has several strengths and limitations. To the best of our knowledge, this is the first large evaluation of a nationwide contraceptive provision programme using a nationally representative sample of women in Argentina. Our study included a large sample size of women who had received the implant through the public health system and data collection was carried out as part of a routine standard of care that included telephone follow-up. However, our study does have some limitations. First, despite the large sample size, we had a low response rate (5.5% among 15–19-year-olds, 10.6% among 20–24-year-olds). Second, the fact that this was a phone survey means that those without access to phones could not be reached, potentially excluding the most vulnerable women. And third, those who administered the survey were also those who delivered the services, possibly contributing to some reticence in providing negative feedback.

## Conclusion

Improving access and uptake of contraception is a critical element of an overall response towards reducing unintended adolescent pregnancy. In preventing unintended pregnancy, it meets the needs and fulfils the rights of adolescents. This study has shown that providing the contraceptive implant as one of the many methods available to women is feasible, acceptable, and effective. This augurs well for Argentina and elsewhere in Latin America and can be extrapolated to other regions of the world.

## Data Availability

The authors bring evidence on the adherence to the implant among adolescents and youth when unintended pregnancy during adolescence is a priority in terms of sexual and reproductive health and rights. Evidence from this study offers guidelines and recommendations that can be extrapolated to similar countries in the Latin American region.

## Footnotes

* Long-acting forms of reversible contraception, i.e. intrauterine devices and contraceptive implants.^33^

## References

[1] World Health Organization. Adolescent pregnancy [Internet]. 2020 [cited 2021 Nov 29]. Available from: https://www.who.int/news-room/fact-sheets/detail/adolescent-pregnancy.

[2] United Nations Department of Economic and Social Affairs. World Population Prospects 2019 [Internet]. 2019 [cited 2021 Nov 29]. Available from: https://population.un.org/wpp/DataQuery/.

[3] Ponce de Leon RG, Ewerling F, Serruya SJ, et al. Contraceptive use in Latin America and the Caribbean with a focus on long-acting reversible contraceptives: prevalence and inequalities in 23 countries. Lancet Glob Heal. 2019 Feb;7(2):e227–e235. 10.1016/S2214-109X(18)30481-9 PMC6367565 30683240

[4] World Health Organization. Maternal mortality: Levels and trends. 2019 [cited 2021 Nov 29]; Available from: http://www.who.int/reproductivehealth/publications/maternal-mortality-2000-2017/en/.

[5] Ministerio de Salud Argentina. Estadísticas vitales. Información Básica. Argentina - Año 2018. Buenos Aires; 2019.

[6] Dirección de Estadísticas e Información en Salud (DEIS). No Title. Ministerio de Salud Argentina, editor. 2019.

[7] Parks C, Peipert JF. Eliminating health disparities in unintended pregnancy with long-acting reversible contraception (LARC). Am J Obstet Gynecol. 2016 Jun;214(6):681–688. 26875950 10.1016/j.ajog.2016.02.017 PMC4884485

[8] Bahamondes L, Bottura BF, Bahamondes MV, et al. Estimated disability-adjusted life years averted by long-term provision of long acting contraceptive methods in a Brazilian clinic. Hum Reprod. 2014 Oct;29(10):2163–2170. 25085802 10.1093/humrep/deu191

[9] Curtis KM, Peipert JF. Long-acting reversible contraception. N Engl J Med. 2017 Feb;376(5):461–468. 28146650 10.1056/NEJMcp1608736 PMC11283813

[10] McNicholas C, Madden T, Secura G, et al. The contraceptive CHOICE project round up: what we did and what we learned. Clin Obstet Gynecol. 2014 Dec;57(4):635–643. 25286295 10.1097/GRF.0000000000000070 PMC4216614

[11] Ricketts S, Klingler G, Schwalberg R. Game change in Colorado: widespread use of long-acting reversible contraceptives and rapid decline in births among young, low-income women. Perspect Sex Reprod Health. 2014 Sep;46(3):125–132. 24961366 10.1363/46e1714

[12] Diedrich JT, Klein DA, Peipert JF. Long-acting reversible contraception in adolescents: a systematic review and meta-analysis. Am J Obstet Gynecol. 2017 Apr;216(4):364.e1–364.e12. 10.1016/j.ajog.2016.12.024 28038902

[13] Dirección General de Población - RENAPER. La natalidad y fecundidad en Argentina entre 1980 y 2019 [Internet]. Buenos Aires: Min. del Interior; 2021 [cited 2021 Nov 29]. Available from: https://www.argentina.gob.ar/sites/default/files/2021/09/natalidad_y_fecundidad_en_argentina.1980_a_2019.dnp_resumen_ejecutivo_nacional_final.pdf.

[14] Carpinetti E, Martínez R. Tendencias recientes y características de la fecundidad adolescente en la ciudad de Buenos Aires. Población de Buenos Aires. 2017 [cited 2021 Nov 29];14(25):51–67. Available from: https://www.redalyc.org/journal/740/74051020003/html/.

[15] Binstock G. Fecundidad y Maternidad Adolescente en el Cono Sur: Apuntes para la Construcción de una Agenda Común. Panama: UNFPA; 2016 [cited 2021 Nov 29]; Available from: https://argentina.unfpa.org/es/publications/fecundidad-y-maternidad-adolescente-en-el-cono-sur-apuntes-para-la-construcción-de-una.

[16] Oizerovich S, Perrota G, Suárez N, et al. Estudio de seguimiento y adherencia al implante subdérmico en adolescentes y jóvenes en Argentina. Buenos Aires: Ministry of Health and Social Development Argentina; 2018 [cited 2021 Nov 29]. Available from: http://iah.salud.gob.ar/doc/Documento214.pdf

[17] Luciana K, Palazzesi A, Ramírez MC. A 12 años de la creación del Programa Nacional de Salud Sexual y Procreación Responsable ¿Cómo estamos? AMAda (Asociación Médica Argentina de Anticoncepción) [Internet]. 2015 [cited 2021 Nov 29];12(2):6–16. Available from: http://www.amada.org.ar/images/revista_Amada_N2_2015.pdf.

[18] Pichardo M, Arribas L, Coccio E, et al. IUDs as EC? limited awareness and high reported acceptability: evidence from Argentina. Contraception. 2014 Nov;90(5):522–528. 24973905 10.1016/j.contraception.2014.05.012

[19] Silva-Filho Ad, Lira J, Rocha ALL, et al. Non-hormonal and hormonal intrauterine contraception: survey of patients’ perceptions in four Latin American countries. Eur J Contracept Reprod Heal Care. 2016 May 3;21(3):213–219. Available from: doi:10.3109/13625187.2015.1137281 26848851

[20] Real JP, De Santis M, Correa Salde V, et al. Perfil de consumo de anticonceptivos orales en la ciudad de Córdoba, Argentina. Ars Pharm. 2014;55(2):32–41.

[21] Rall P, Dejean L, Luna M, et al. Aceptabilidad del implante subdérmico y el perfil sociodemográfico de sus usuarias, durante el primer año de uso. Rev AMAdA (Asociación Médica Argentina Anticoncepción). 2017 [cited 2021 Nov 29];13(2):4–14. Available from: http://www.amada.org.ar/index.php/revista/numeros-anteriores/volumen-13-n-2-2017/202-aceptabilidad-del-implante-subdermico-y-el-perfil-sociodemografico-de-sus-usuarias-durante-el-primer-ano-de-uso.

[22] Programa Nacional de Salud Sexual y Procreación Responsable Ministerio de Salud, Instituto Nacional de Estadística y Censos - INDEC. Encuesta nacional sobre salud sexual y reproductiva [Internet]. Buenos Aires; 2014 [cited 2021 Nov 29]. Available from: https://bancos.salud.gob.ar/sites/default/files/2018-10/0000000729cnt-encuesta_nacional_sobre_salud_sexual_y_reproductiva.pdf.

[23] Harvey C, Seib C, Lucke J. Continuation rates and reasons for removal among Implanon users accessing two family planning clinics in Queensland, Australia. Contraception. 2009 Dec;80(6):527–532. 19913146 10.1016/j.contraception.2009.05.132

[24] Kalmuss D, Davidson AR, Cushman LF, et al. Determinants of early implant discontinuation among low-income women. Fam Plann Perspect. 1996;28(6):256–260. 8959415

[25] Trussell J. Contraceptive efficacy. In: Hatcher RA, Trussell J, Nelson AL, Cates W, Kowal D, Policar M, editors. Contraceptive technology: Twentieth revised edition. New York: Ardent Media; 2011;397–404.

[26] Bahamondes L, Brache V, Meirik O, et al. A 3-year multicentre randomized controlled trial of etonogestrel- and levonorgestrel-releasing contraceptive implants, with non-randomized matched copper-intrauterine device controls. Hum Reprod. 2015 Nov;30(11):2527–2538. 26409014 10.1093/humrep/dev221

[27] Modesto W, Bahamondes MV, Bahamondes L. A randomized clinical trial of the effect of intensive versus non-intensive counselling on discontinuation rates due to bleeding disturbances of three long-acting reversible contraceptives. Hum Reprod. 2014 Jul;29(7):1393–1399. 24812309 10.1093/humrep/deu089

[28] Rubenstein J, Rubenstein P, Barter J, et al. Counselling styles and their effect on subdermal contraceptive implant continuation rates. Eur J Contracept Reprod Heal Care Off J Eur Soc Contracept. 2011 Jun;16(3):225–228. 10.3109/13625187.2011.561939 21395387

[29] Davie JE, Walling MR, Mansour DJ, et al. Impact of patient counseling on acceptance of the levonorgestrel implant contraceptive in the United Kingdom. Clin Ther. 1996;18(1):150–159. 8851460 10.1016/s0149-2918(96)80187-1

[30] Luchowski AT, Anderson BL, Power ML, et al. Obstetrician-gynecologists and contraception: long-acting reversible contraception practices and education. Contraception. 2014 Jun;89(6):578–583. 24656553 10.1016/j.contraception.2014.02.004

[31] Bahamondes L, Villarroel C, Frías Guzmán N, et al. The use of long-acting reversible contraceptives in Latin America and the Caribbean: current landscape and recommendations. Hum Reprod Open. 2018 Jan 23;2018(1):hox030–hox030. Available from: https://pubmed.ncbi.nlm.nih.gov/30895242. 30895242 10.1093/hropen/hox030 PMC6276683

[32] Saavedra-Avendano B, Andrade-Romo Z, Rodriguez MI, et al. Adolescents and long-acting reversible contraception: lessons from Mexico. Matern Child Health J. 2017 Sep;21(9):1724–1733. 27150948 10.1007/s10995-016-2013-1 PMC5569121

[33] Darney BG, Fuentes-Rivera E, Saavedra-Avendaño B, et al. Contraceptive receipt among first-trimester abortion clients and postpartum women in urban Mexico. Int Perspectives Sexual Reprod Health. 2020 Dec 1;46(Supplement 1):35–43. 10.1363/46e0720 33326398

[34] World Health Organization. The Global Strategy for Womeńs, Childreńs and Adolescentś Health (2016-2030) [Internet]. 2015 [cited 2021 Nov 30]. Available from: https://www.who.int/life-course/partners/global-strategy/globalstrategyreport2016-2030-lowres.pdf.

[35] Committee on Practice Bulletins-Gynecology Long-Acting Reversible Contraception Work Group. Practice bulletin No. 186: long-acting reversible contraception: implants and intrauterine devices. Obstet Gynecol. 2017 Nov;130(5):e251–e269. 29064972 10.1097/AOG.0000000000002400

